# Si-Ni-San improves the deposition of lipid droplets in MAFLD through modulating the FXR-GPAT4 axis

**DOI:** 10.1186/s13020-025-01309-5

**Published:** 2026-01-13

**Authors:** Haibo Fan, Yalei Hou, Yue Li, Zhiwen Zheng, Xuelun Wang, Yunfeng Li, Yongmin Li

**Affiliations:** 1 Hebei University of Chinese Medicine, No.326 Xinshi South Road, Qiaoxi District, Shijiazhuang, 050200 Hebei China; 2 Hebei Key Laboratory of Health Care with Traditional Chinese Medicine, Shijiazhuang, 050200 China; 3 The Seventh People’s Hospital of Hebei Province, Dingzhou, 073000 China; 4 Hebei Key Laboratory of Chinese Medicine Research on Cardio-Cerebrovascular Disease, No.326 Xinshi South Road, Qiaoxi District, Shijiazhuang, 050091 Hebei China

**Keywords:** Metabolic-associated fatty liver disease, Si-Ni-San, Farnesoid X receptor, Glycerol 3-phosphate acyltransferase 4, Molecular dynamics simulations

## Abstract

**Background:**

Metabolic-associated fatty liver disease (MAFLD) is a common metabolic disease with complex pathogenesis and lack of effective treatment. Si-Ni-San (SNS), a traditional Chinese medicine, has emerged as a promising candidate for MAFLD treatment. However, the protective mechanism remains unclear.

**Methods:**

C57BL/6N mice were fed with high-fat diet (HFD) for 12 weeks to establish MAFLD mouse model. Concurrently, oleic acid-induced HepG2 cells were used in vitro as a cellular model for MAFLD. The effects of SNS and the positive drug obeticholic acid on hepatic lipid droplets deposition in MAFLD mice and cell models were evaluated. The expression levels of farnesoid X receptor (FXR) and glycerol 3-phosphate acyltransferase 4 (GPAT4) were detected by western blot. The siRNA and dual-luciferase reporter assay were used to detect the interaction between FXR and GPAT4. High-performance liquid chromatography (HPLC) was used to identify the active components in the SNS aqueous solution, and their binding affinities to targets were detected through molecular docking, molecular dynamics simulations, and surface plasmon resonance (SPR).

**Results:**

The active ingredients of SNS were identified by HPLC. SNS ameliorated hepatic lipid droplets deposition in both mouse and cellular models of MAFLD. SNS up-regulated the expression of FXR and down-regulated the expression of GPAT4 in hepatic tissues, thereby modulating proteins involved in hepatic lipolysis and lipophagy. FXR reduced lipid droplets accumulation by inhibiting GPAT4. The dual-luciferase reporter assay confirmed that FXR transcriptionally regulated and inhibited GPAT4 expression. Furthermore, molecular docking and molecular dynamics simulations predicted potential interactions between the active components of SNS and the FXR and GPAT4 proteins, with the binding affinity for FXR being subsequently confirmed through SPR analysis.

**Conclusion:**

This study provided a new mechanistic exploration for FXR in improving MAFLD and broadened the research direction on the mechanisms by which SNS reduced hepatic lipid droplets deposition. It also offers a molecular dynamics basis for subsequent studies on how active components in SNS exert their effects through binding to FXR.

## Introduction

Metabolic-associated fatty liver disease (MAFLD) is an excessive accumulation of fat in the liver that associated with metabolic dysfunction and insulin resistance in the developing of overweight or obesity [1]. In 2020, an international consensus changed non-alcoholic fatty liver disease (NAFLD) to MAFLD [2]. MAFLD could progress to metabolic dysfunction-associated steatohepatitis (MASH), liver cirrhosis, and hepatocellular carcinoma [3]. The global incidence rate of MAFLD is 25%, which has led to high costs for the global health system. Approximately 1.7 billion people have suffered from MAFLD [4]. By 2030, MAFLD will affect about 33.5% of the adult population, and MASH will affect about 27% of cases [5]. Currently, the "multiple hits" theory accepted by most researchers is the combined result of chronic inflammation, oxidative stress, epigenetics, and genetic susceptibility factors such as obesity, high-fat diet (HFD) intake, and insulin resistance (IR) [6]. Due to the complex pathogenesis of MAFLD, there are currently no approved effective therapeutic drugs [7]. Therefore, this study explored the efficacy and mechanism of traditional Chinese medicine (TCM) in the prevention and treatment of MAFLD.

Bile acids (BAs) are bioactive molecules synthesized in the liver and secreted into the intestine through the bile duct [8]. BAs may have great potential as a therapeutic breakthrough in the treatment of MAFLD. Farnesol X receptor (FXR) is a key endogenous receptor of BAs, and it mainly expresses in hepatocytes and intestinal epithelial cells [9]. In the liver, FXR reduces hepatic lipids through the small heterodimer mate (SHP)-sterol regulatory element-binding protein 1c (SREBP1c) pathway-mediated hepatic de novo lipogenesis [10]. Additionally, FXR activation enhances the transcriptional activity of peroxisome proliferator-activated receptor α (PPARα), promoting fatty acid β-oxidation [11]. In summary, BAs homeostasis is a key point for improving MAFLD, and FXR is widely recognized as a landmark target in the MAFLD field.

Lipid droplets (LDs) are important subcellular organelles that maintain lipid homeostasis by coordinating lipid synthesis, lipid storage, and lipid secretion, and they are surrounded by phospholipid monolayers around the lipid core. Two major pathways mediate LDs protein targeting. Glycerol 3-phosphate acyltransferase 4 (GPAT4) is involved in one pathway, the endoplasmic reticulum (ER)-LDs targeting, and GPAT4 is transported to LDs through the ER-LDs membrane bridge [12]. GPAT4 is predominantly distributed in hepatocytes and expressed only in the ER. At the subcellular level, GPAT4 could be transported from the ER to the LDs and promote the growth of the LDs [13]. Studies have demonstrated that GPAT4^−/−^ mice exhibited a 25% reduction in body weight and are resistant to both diet-induced and hereditary obesity. These animals also showed a 45% decrease in total hepatic GPAT activity and triglyceride (TG) content [14]. However, it remains unclear whether hepatic FXR could modulate GPAT4 to ameliorate lipid accumulation.

Lipolysis and lipophagy play crucial roles in lipid metabolism and are closely associated with MAFLD. Lipolysis, mediated by enzymes such as adipose triglyceride lipase (ATGL), monoacylglycerol lipase (MAGL), and hormone-sensitive lipase (HSL), breaks down triglycerides stored in adipocytes, releasing fatty acids for energy utilization [15]. In MAFLD, impaired lipolysis could lead to excessive lipid accumulation in the liver. Lipophagy, a selective form of autophagy related to lipid metabolism, involves proteins like sequestosome 1 (P62), Beclin1, and microtubule-associated protein 1 light chain 3 (LC3). P62 acts as a linker molecule, recruiting LDs to the autophagosome formation site. Beclin1 initiates the autophagy process, and LC3 is conjugated to the autophagosome membrane, facilitating lipid degradation [16]. Dysfunction in lipophagy could disrupt the balance of lipid turnover in the liver, contributing to the progression of MAFLD. Several studies have shown that in MAFLD models, decreased expression of ATGL and abnormal activation of P62-mediated lipophagy were observed, leading to aggravated hepatic steatosis [17].

TCM has certain advantages in the treatment of MAFLD [7]. Clinically, most patients present with symptoms, such as distending pain in the hypochondrium, abdominal fullness, fatigue, light red tongue, and white greasy coating of the tongue. Clinical studies have shown that regulating the metabolism of liver and spleen is an effective treatment for MAFLD patients [18]. Si-Ni-San (SNS), a TCM formulation, has been used in China for centuries. Initially documented by the renowned Han Dynasty physician Zhang Zhong-jing in his work *Shang Han Lun*, it consists of four herbs in equal proportions: Bupleuri Radix (*Bupleurum chinense* DC., CH), Paeoniae Radix Alba (*Paeonia lactiflora* Pall., BS), Aurantii Fructus Immaturus (*Citrus trifoliata* l., ZS), and Glycyrrhizae radix et Rhizoma Praeparata Cum Melle (*Glycyrrhiza uralensis* Fisch., GC) [19]. Our previous work demonstrated that SNS reduced LDs deposition in MAFLD and confirmed that SNS could improve LDs deposition by inhibiting Yes-associated protein 1 (YAP1) expression [20, 21]. The present study investigated the relationship between FXR and GPAT4, and the mechanism by which SNS improves LD deposition in MAFLD.

## Materials and methods

### Preparation of the SNS decoction

Following our previous study [21], equal masses of bupleurum, peony, bitter orange, and licorice (sourced from the National Medical Hall of Hebei University of Traditional Chinese Medicine) were mixed in a 1:1:1:1 ratio and decocted twice. The two resulting solutions were combined, concentrated to 100 mL, and stored at 4 °C.

### Prepare obeticholic acid (OCA) for animals and cells

Obeticholic acid (OCA) was obtained from Shanghai Aladdin Biochemical Technology Co., Ltd. For the animal experiments, OCA was dissolved in corn oil to prepare a 10 mg/kg solution for intragastric administration to mice. For the cell experiments, OCA was dissolved in dimethyl sulfoxide (DMSO) to achieve a final concentration of 1 μM.

### Animal study

All the animal experiments adhere to National Guidelines for the Management and Use of Laboratory Animals, and received approval of Animal Ethics Committee of Hebei University of Chinese Medicine on March 5, 2024, with the ethical approval number DWLL202403092. Forty male C57BL/6N mice aged 6–7 weeks, specific pathogen-free (SPF) grade, were purchased from Changsheng Biotechnology Co., Ltd. (Liaoning, China). The mice were housed in a temperature-controlled environment with a 12-h light/dark cycle.

After a 1-week acclimation period, the mice were randomly assigned to four groups (n = 10): normal control group (NC), high-fat diet group (HFD), HFD and SNS group (SNS), HFD and OCA group (OCA). A standard diet was fed to the NC group, while HFD (D12451, Huanyu Hekang Biotechnology, China) was fed to the other four groups for 12 weeks. From the 10th week of the experiment, the mice of the SNS and OCA groups were orally given SNS (5.2 g/kg/d, 0.1 mL/mouse) or OCA (10 mg/kg/d) for 2 weeks. During this period, mice in the NC group and HFD group were given isovolumetric distilled water respectively. As shown in Fig. 2A, after the experiment, the body and liver weights of the mice were measured, and the liver-to-body weight ratio (liver weight/body weight) was calculated.

### Preparation of SNS-containing serum

Healthy male Sprague–Dawley (SD) rats (5–6 weeks old, weighing 180 ± 10 g) were obtained from Changsheng Biotechnology Co., Ltd. (Liaoning, China). After a 1-week acclimation period, the rats were administered SNS decoction (3.6 g/kg) or normal saline (NS) in equal volumes daily for 1 week [22]. Blood was collected from the abdominal aorta after the rats were anesthetized with 1% pentobarbital (0.01 mL/g). The serum was isolated, collected, inactivated at 56 °C, and stored at −80 °C for later use in cell experiments as containing serum. The SNS-containing serum and NS-containing serum were derived from the SNS-treated and NS-treated mice, respectively.

### High performance liquid chromatography (HPLC)

The SNS formulation contained equal masses (6 g each) of Paeoniae Radix Alba (Baishao), Bupleuri Radix (Chaihu), Aurantii Fructus Immaturus (Zhishi), and Glycyrrhizae Radix et Rhizoma Praeparata (Zhigancao). Botanical materials were hydrated in tenfold distilled H₂O (v/w) for 60 min, followed by sequential extraction: primary decoction at vigorous boiling (100 °C, 20 min), secondary extraction at gentle simmering (90 °C, 40 min). The combined filtrates were gauze-filtered and concentrated to 70 mg/mL via rotary evaporation.

The qualitative analysis of SNS was performed on an Agilent Series 1260 Infinity HPLC system (Agilent Technologies Inc., USA) equipped with a Zorbax StableBond-AQ C18 column (250 × 4.6 mm, 5 μm) (Agilent Technologies Inc, USA) maintained at 30 °C. The mobile phase consisted of acetonitrile solvent A and water with 0.1 phosphoric acid solvent B, and the following linear gradient elution procedure was used: 0–5 min, 5–10% A; 5–14 min, 10–12% A; 14–25 min, 12–18% A; 25–50 min, 18–23.5% A; 50–55 min, 23.5–28.5% A; 55–70 min, 28.5–36% A; 70–75 min, 36–40% A; 75–85 min, 40–44% A; 85–90 min, 44–90% A. This analysis was performed at a wavelength of 240 nm with a flow rate of 1 mL/min and an injection volume of 10 μL.

### The serum biochemical assays

Serum samples were collected to measure the content of TG, total cholesterol (TC), alanine aminotransferase (ALT), aspartate aminotransferase (AST), high-density lipoprotein cholesterol (HDL-C), and low-density lipoprotein cholesterol (LDL-C). The Reado/Changchun Huili kit was used for the assay, measured by a fully automatic biochemistry analyzer.

### Total bile acid (TBA) content analysis

The TBA content in liver tissue was detected by a TBA assay kit (E003-2-1, Nanjing Jiancheng Bioengineering Institute, China). According to the kit instruction manual, the liver tissues were subjected to lysis, after which the supernatant was collected and added to a 96-well plate. Blank controls and standard controls were set up. Subsequently, 180 μL of Reagent 1 was added, followed by gentle shaking to mix thoroughly, and incubation at 37 °C for 5 min. After that, 60 μL of Reagent 2 was added, mixed well by gentle shaking, and incubated at 37 °C for 1 min. The absorbance (A0) was measured at a wavelength of 405 nm using a microplate reader. Another incubation at 37 °C for 3 min was conducted, and then the absorbance (A1) was measured again. The ΔA value was calculated as ΔA = A1−A0, which represents the TBA content in the liver tissues.

### Cell culture

The HepG2 cell line was purchased from the American Type Culture Collection (Manassas, USA) and cultured in RPMI 1640 medium (Solarbio, China) with 10% fetal bovine serum (Gibco, USA) in an atmosphere with 5% CO_2_ and 100% humidity at 37 ℃. Oleic acid (OA) was purchased from Shanghai Macklin Biochemical Co., Ltd. (C11391320, Macklin, China). For incubation, the stock solutions of OA (100 μM) were prepared in isopropanol at a concentration of 20 mM and then temporarily diluted in RPMI 1640 medium with 10% fetal bovine serum, so that the final concentration of the isopropanol did not exceed 1% [23]. Cells were inoculated into 6-well plates and 96-well plates and treated with OA solution and SNS/NS-containing serum for 24 h. Based on our previous study, a 15% SNS-containing serum concentration was selected for the in vitro experiments, as it did not affect cell viability [20].

### Hematoxylin and eosin staining (H&E)

Liver tissue from each group of mice was fixed in 4% formalin, embedded in paraffin, and prepared as paraffin tissue sections with a thickness of 5 μm. The sections were dewaxed and rehydrated, subsequently subjected to H&E staining, and finally examined under a microscope (Leica DM2500, Germany) for the observation of LDs.

### Oil red O staining

Lipid accumulation in vivo was assessed by oil red O (ORO) staining (G1261, Solarbio, China). For liver tissue, frozen tissue sections were prepared at a thickness of 10 μm. After fixation in formalin, the sections were washed in 60% isopropanol and then stained with freshly prepared ORO working solution for 15 min. Following rinsing with 60% isopropanol, the sections were examined under a microscope.

In vitro*,* the HepG2 cell cells were firstly seeded in 6-well plates (3 × 10^5^ cells/well). After the cells were washed with PBS, ORO fixative was added to each well for 20–30 min, and 60% isopropyl alcohol was added to each well for 5 min. Then, ORO staining solution was used to stain the cells for 10–20 min. After the cells were washed with PBS, hematoxylin staining solution was used to re-stain the nucleus. Then, we added ORO buffer for 1 min. Finally, we used glycerin gelatin to seal the tablets. The changes in LDs were measured with Image Pro Plus v6.0 software (Media Cybemetic, USA).

### Quantitative real-time PCR (qRT-PCR)

Total RNA was extracted from cells and reverse transcribed into complementary DNA (cDNA) for the assessment of gene expression levels. These reactions were amplified in a LightCyclerR 480 Quantitative PCR System (Roche, USA), and the resulting data were analyzed using the 2^−ΔΔCt^ method. This enabled the determination of transcript expression levels by normalizing to the expression levels of a housekeeping gene. The primer sequences used in this study are shown in Table 1.

**Table 1:** Sequences of qPCR primers of human

Gene name		Sequence (5’ to 3’)
*FXR*	Forward	ACTTCCGTCTGGGCATTCTGAC
	Reverse	GCTGTAAGCAGAGCATACTCCTC
*GPAT4*	Forward	ACTGGCTTTCACAGGGATTAG
	Reverse	CGCAGATCCGGTAACACATTA
*GAPDH*	Forward	ATCCCATCACCATCTTCC
	Reverse	CCATCACGCCACAGTTC

### Western blot

Total protein was extracted using a protein extraction kit (PC201Plus, Epizyme). Then, the protein concentration was measured using a BCA kit (PC0020, Solarbio). The 50 μg of the total protein samples were loaded onto an SDS-PAGE gel. Following electrophoresis, the protein was transferred into polyvinylidene fluoride (PVDF) membranes. Whereafter, the membranes were first blocked with 5% nonfat dry milk in TBST and incubated with primary antibodies against FXR (bs-12867R, Bioss, 1:1000), GPAT4 (bs-15587R, Bioss, China, 1:1000), P62 (380612-50, Zenbio, China, 1:1000), HSL (344379-50, Zenbio, China, 1:1000), MAGL (PAD223Hu01, Cloud-Clone Corp, China, 1:1000), Beclin1 (BS-1353R, Bioss, China, 1:1000), ATGL (R389129-50, Bioss, China, 1:1000), LC3Ⅰ/Ⅱ (ABC929, Merck, USA, 1:1000), β-actin (bs-0061R, Bioss, China, 1:5000) and GAPDH (GB11002-100, Servicebio, China, 1:5000). After 12 h of incubation at 4 °C, the blots were proceeded with HRP-conjugated secondary antibody. Finally, the immune bands were detected using an ECL detection kit (sb-wb012, Shanghai Shenger Biotechnology Co., Ltd, China). The average gray value was quantified using Image J software.

### Immunohistochemistry assay (IHC)

The paraffin sections of liver were performed dewaxing followed by antigen retrieval, then add FXR primary antibody (bs-12867R, Bioss, 1:200) and GPAT4 primary antibody (bs-1924R, Bioss, 1:200), and incubate overnight at 4 °C. After the diaminobenzidine (DAB) enhanced staining, the sections were mounted with neutral gum. Finally, the images were observed and captured via a Leica DM4000B light microscope (Germany).

### RNA interference

Small interfering RNA (siRNA)-to knock down FXR (si-FXR), GPAT4 siRNA (si-GPAT4), and negative control NC (si-NC) were designed and synthesized by GENEPHARMA (Jiangsu, China). HepG2 cell lines were transfected with the siRNAs using siRNA mate plus (G04026, GENEPHARMA, China). Cells were seeded in complete medium approximately 12 h before transfection. Then, siRNAs mixed with siRNA mate plus were added to the cells with fresh Opti-MEM medium (Gibco, USA). SiRNAs were transfected at a concentration of 50 nM. After 6 h, the medium containing siRNAs and siRNA mate plus was replaced with complete medium. The sequences of siRNAs are shown in Table 2.

**Table 2:** The sequences of siRNAs of human

Name	Forward	Reverse
*FXR-homo-651*	CCACAGAUUUCCUCGUCAUTT	AUGACGAGGAAAUCUGUGGTT
*FXR-homo-786*	GCAGAGAUGCCUGUAACAATT	UUGUUACAGGCAUCUCUGCTT
*FXR-homo-877*	CCUCUGGAUACCACUAUAATT	UUAUAGUGGUAUCCAGAGGTT
*GPAT4-Homo-1362*	GCUGAGCAGAACCAAUUAUTT	AUAAUUGGUUCUGCUCAGCTT
*GPAT4-Homo-1505*	GCACAACUGUGGUGGGAUATT	UAUCCCACCACAGUUGUGCTT
*GPAT4-Homo-1965*	GCACAACUGUGGUGGGAUATT	AUACUUGAUAGCAACAGGGTT

### Database prediction and screening

Analyze the correlation and binding sites between the GPAT4 promoter region and the transcription factor FXR using the JASPAR database (www.jaspar.elixir.no). Predict and screen the transcription factors of GPAT4 using the UCSC Genome Browser (www.genome.ucsc.edu) and obtain the binding score of FXR and GPAT4.

### Dual-luciferase reporter assays (DL)

Six plasmids (pcDNA3.1(+)-FXR-3xFLAG, pcDNA3.1(+)-MCS-3xFLAG, pGL4.10-GPAT4 promoter (WT), pGL4.10, pGL4.10-GPAT4 promoter (MUT), pRL-CMV) were purchased from Obio Technology Corp (Shanghai, China) for the interaction of FXR and GPAT4-promoter experiment. HepG2 cells were randomly divided into the following four groups: pcDNA3.1(+)-FXR-3Xflag + pGL4.10-GPAT4 promoter (WT); pcDNA3.1(+)-FXR-3xFLAG + pGL4.10-GPAT4 promoter (MUT); pcDNA3.1(+)-MCS-3xFLAG + pGL4.10-GPAT4 promoter (WT); pcDNA3.1(+)-MCS-3xFLAG + pGL4.10-GPAT4 promoter (MUT) Four groups of cells were transfected with the corresponding plasmids and the pRL-CMV plasmid for 24 h. The cells were collected to calculate the ratio of firefly to Renilla luciferase activity using the dual luciferase reporter gene assay kit (Promega, Madison, USA).

### Molecular docking simulations

The 3D structure models of core target proteins were downloaded from the Protein Data Bank (RCSB-PDB, https://www.rcsb.org/) and imported into PyMOL 2.5.2 for dehydration and ligand separation. The 2D structures of ligand small molecules were downloaded from the PubChem database (https://pubchem.ncbi.nlm.nih.gov/), and key components were converted into mol2 format files using ChemBioOffice. AutoDockTools-1.5.7 was used to add non-polar hydrogens to the receptor protein and ligand small molecules, calculate charges for the protein structure, and identify rotatable bonds in the ligand molecules. Appropriate docking boxes and parameters were set according to the structural sizes of the receptor and ligand, and molecular docking was performed using AutoDockTools 1.5.7. Visualization analysis was conducted using PyMOL.

### Molecular dynamics simulation (MD simulation)

In this study, Gromacs 2022 was used for molecular dynamics simulations. Force field parameters were obtained using the pdb2gmx tool in Gromacs and the AutoFF web server. During the simulation, the CHARMM36 force field [24] was applied to the molecular parameters of the receptor protein, while the CGenff force field was used for the ligand molecular parameters. The system was solvated by adding a 1 nm TIP3P cubic water box around it. Ions were added to the system using the GROMACS genion tool to achieve electrical neutrality. Long-range electrostatic interactions were handled by the Particle Mesh Ewald (PME) method with a cutoff distance of 1 nm. All bond constraints were implemented via the SHAKE algorithm, and the Verlet leapfrog algorithm was adopted with an integration time step of 1 fs for the MD simulation process.

Prior to molecular dynamics simulation, the system underwent energy minimization, which included 3000 steps of steepest descent optimization followed by 2000 steps of conjugate gradient optimization. The optimization steps were as follows: first, the solute was constrained, and energy minimization was performed on water molecules; then, the counterions were constrained for energy minimization; finally, energy minimization was conducted on the entire system without constraints.

The simulation was run under the conditions of 310 K and an NPT system at constant pressure, with a total simulation time of 100 ns. During the simulation, root mean square deviation (RMSD), root mean square fluctuation (RMSF), hydrogen bonds (HBonds), radius of gyration (Rg), and solvent-accessible surface area (SASA) were calculated, respectively.

### Surface plasmon resonance (SPR)

SPR analysis was performed by a Biacore S200 system (Cytiva, USA) equipped with a CM5 sensor chip. The FXR protein was immobilized on the chip via the standard amine coupling protocol, with the protein dissolved in a sodium acetate buffer at pH 4.5 and a concentration of 10 mM. Eight candidate compounds, initially prepared at a concentration of 250 μM in a running buffer containing 5% DMSO, were serially diluted to achieve final concentrations as 0.488 μM. Equilibrium dissociation constants (K_D_) were determined by fitting the response curves using the steady-state affinity model available in the Biacore S200 Evaluation Software.

### Statistical analysis

All measurement data are expressed as mean ± standard deviation (mean ± SD). All data were analyzed using SPSS 23.0 software. Normality tests were performed on all data. For data satisfying normal distribution, one-way analysis of variance (ANOVA) was used for comparison. When variances were homogeneous, Turkey's method was applied for pairwise comparisons; when variances were heterogeneous, the Dunnett T3 method was used for pairwise comparisons. The *p* < 0.05 were considered statistically significant. All graphs were created by GraphPad Prism 8.0 software.

## Results

### Qualitative analysis of the major bioactive components of the SNS by HPLC

**Fig. 1:**
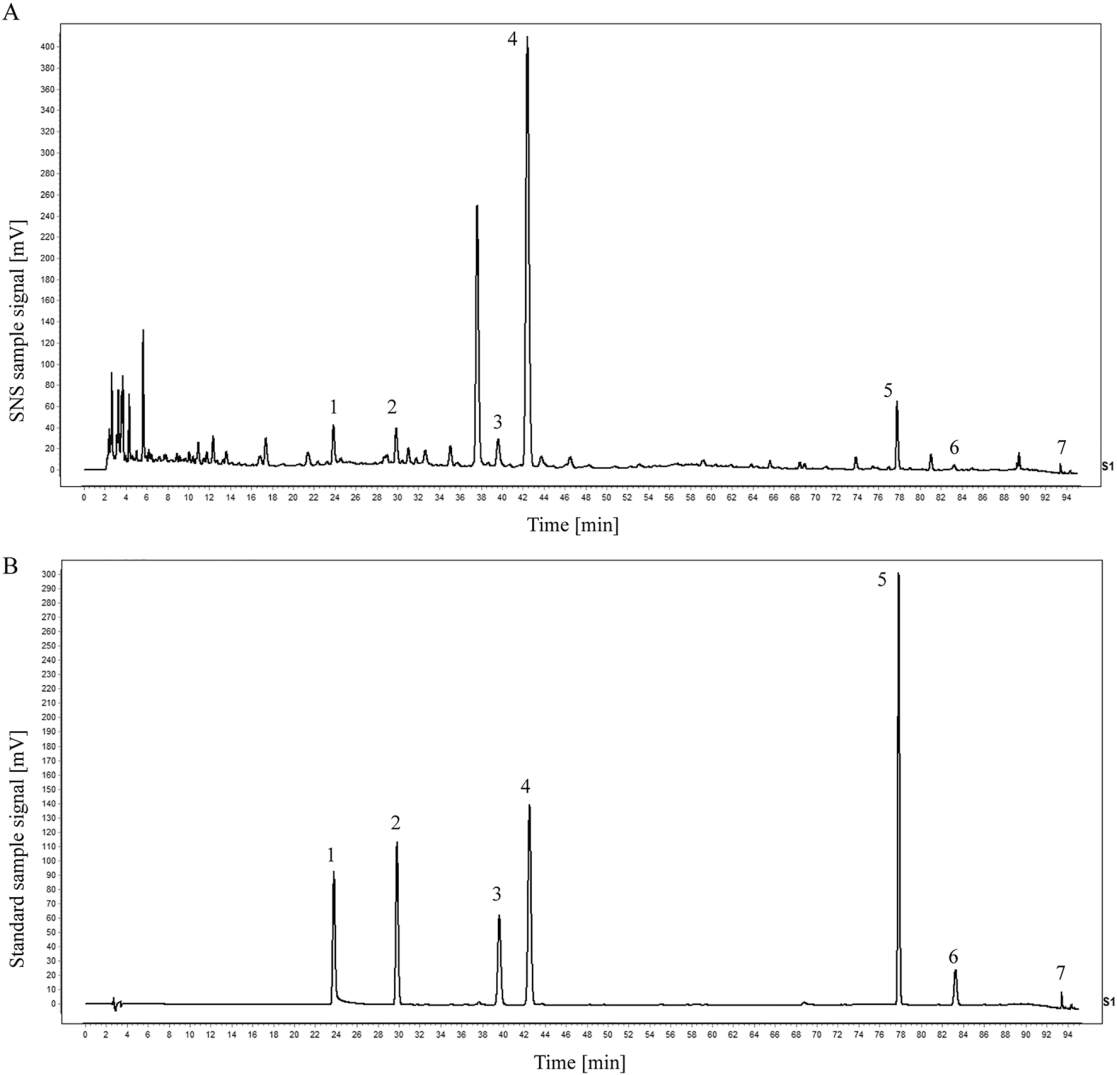
Seven active components in the aqueous decoction of SNS were screened by HPLC. **A** HPLC chromatogram of the concentrated solution of SNS. **B** HPLC chromatogram of the mixed standard solution. Peaks 1, 2, 3, 4, 5, 6, and 7 correspond to the seven active components, respectively

The major components of SNS had been verified by HPLC (Fig. 1A–B). The major bioactive components of SNS, including neohesperidin, peoniflorin, hesperidin, glycyrrhizin, liquiritin, isorhamnetin, and nobiletin were detected and quantified via HPLC (Table 3).

**Table 3:** Seven major active components in the aqueous decoction of SNS

Active ingredient	Peak area	Concentration (mg/mL)
Neohesperidin	12578.7	2.316
Peoniflorin	777.3	0.327
Hesperidin	1107.4	0.202
Glycyrrhizin	1191.5	0.093
Liquiritin	727.6	0.067
Isorhamnetin	96.4	0.005
Nobiletin	122.7	0.002

### SNS reduced the body weight of HFD mice

**Fig. 2:**
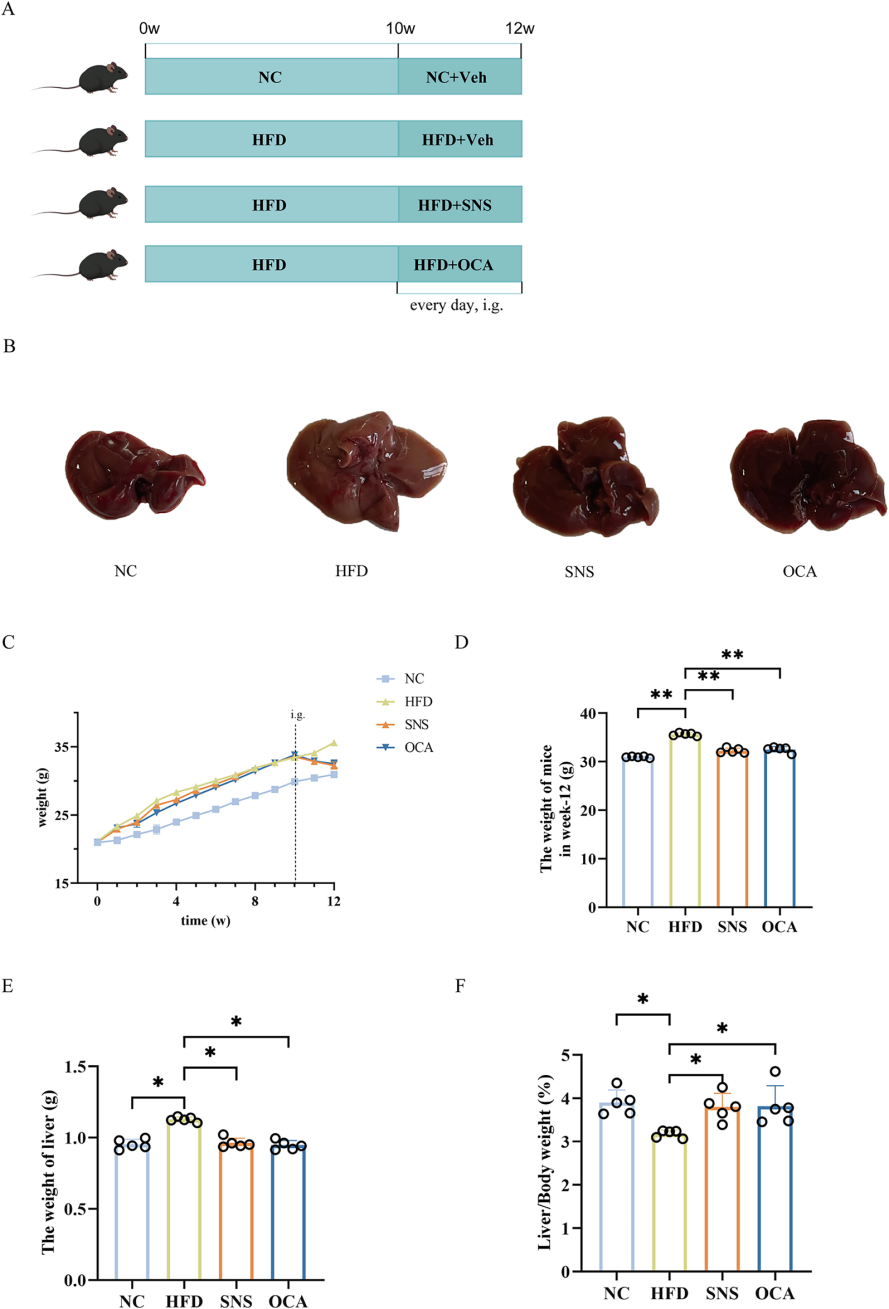
SNS reduces the body weight of HFD mice. **A** Schematic illustration of the animal model construction and drug administration protocol. **B** Representative macroscopic image of liver tissues from each group.** C** Body weight changes were recorded over a 12-week period. **D** Body weights at week 12. **E** Liver weights at the end of the study. **F** Liver-to-body weight ratio. NC, normal control; HFD, high-fat diet; SNS, Si-Ni-San; OCA, obeticholic acid. Data are presented as mean ± SD (n = 5). *p < 0.05, **p < 0.01

To examine the effect of SNS on MAFLD, we developed a mouse model of MAFLD induced by HFD (Fig. 2A). Body weight measurements were taken weekly over a 12-week duration. In the HFD group, there was a consistent increase in body weight, with a statistically significant difference observed in comparison to NC group. Commencing in week 10, treatments with SNS and OCA were initiated, leading to either stabilization or a gradual reduction in body weight (Fig. 2C). By the 12th week, body weights were assessed, revealing that the HFD group had the highest body weight, significantly differing from the NC group. Both SNS and OCA adminstration treatments significantly decreased body weight relative to the HFD group, indicating that SNS and OCA are effective in attenuating body weight gain in MAFLD-afflicted mice (Fig. 2D).

In our study, we observed that the hepatic pathological structure of mice subjected to HFD exhibited enlargement, a yellowish discoloration, and a hardened texture. In contrast, the mice in the SNS and OCA treatment groups closely resembled those in the NC group (Fig. 2B). Subsequent measurements of liver weights indicated that treatment with SNS and OCA resulted in a significant reduction in liver weights in mice with MAFLD induced by a HFD (Fig. 2E). Additionally, the liver coefficient, defined as the ratio of liver weight to body weight, was calculated (Fig. 2F). The findings revealed that the liver coefficient in the HFD group was significantly lower than that in the NC group, whereas the coefficients in the SNS and OCA groups were restored to normal levels. Overall, these phenotypic observations demonstrated that both SNS and OCA effectively ameliorated MAFLD, exhibiting comparable therapeutic efficacy.

### SNS improved liver LDs deposition in MAFLD mice

**Fig. 3:**
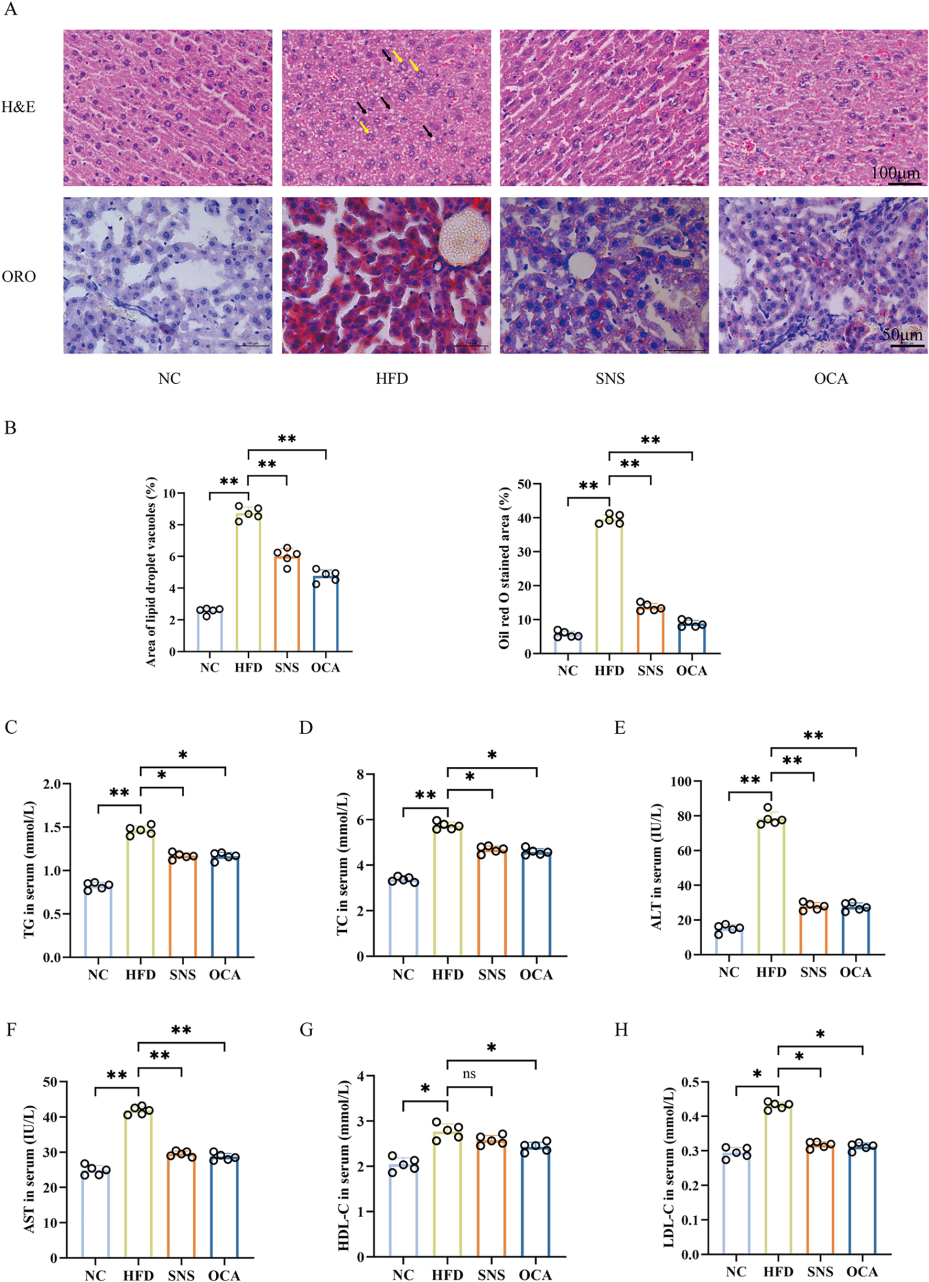
SNS improved liver LDs deposition in MAFLD mice. **A**, **B** Representative H&E and ORO staining images of liver sections from each group, accompanied by the quantification and statistical analysis. LDs vacuoles are indicated by black arrows, and hepatocyte ballooning degeneration is indicated by yellow arrows (n = 5). **C** Serum TG. **D** Serum TC. **E** Serum ALT. **F** Serum AST. **G** Serum HDL-C. **H** Serum LDL-C. Scale bar, 50 μm and 100 μm. Data are expressed as mean ± SD (n = 5), with statistical significance indicated as *p < 0.05 and **p < 0.01

To investigate the mechanisms by which SNS and OCA alleviate MAFLD, we further examined liver LDs deposition. H&E and ORO staining revealed an increase in LDs vacuoles and ORO-positive areas in the HFD group compared to the NC group. In contrast, the SNS and OCA groups exhibited significantly reduced LDs compared to the HFD group, indicating that both SNS and OCA effectively alleviated LDs accumulation (Fig. 3A–B). Serum analysis showed changes in the levels of TC, TG, HDL-C, LDL-C, ALT, and AST. Lipid parameters, including TG, TC, and LDL-C, were elevated in the HFD group but reduced following SNS and OCA treatment. However, SNS had a limited effect on HFD-induced increases in HDL-C levels, while its effects on other parameters were comparable to OCA (Fig. 3C–D, G–H). Serum ALT and AST are markers of liver injury. Significantly elevated ALT and AST were observed in the HFD group, while SNS and OCA treatment effectively reduced liver injury induced by HFD, suggesting that the drugs do not cause liver injury in mice (Fig. 3E–F). Histopathological analysis and serum biochemical assays showed that SNS and OCA improved hepatic LDs deposition and liver injury in MAFLD mice.

### SNS activated FXR to regulate lipolysis- and lipophagy-related protein expression to improve MAFLD

**Fig. 4:**
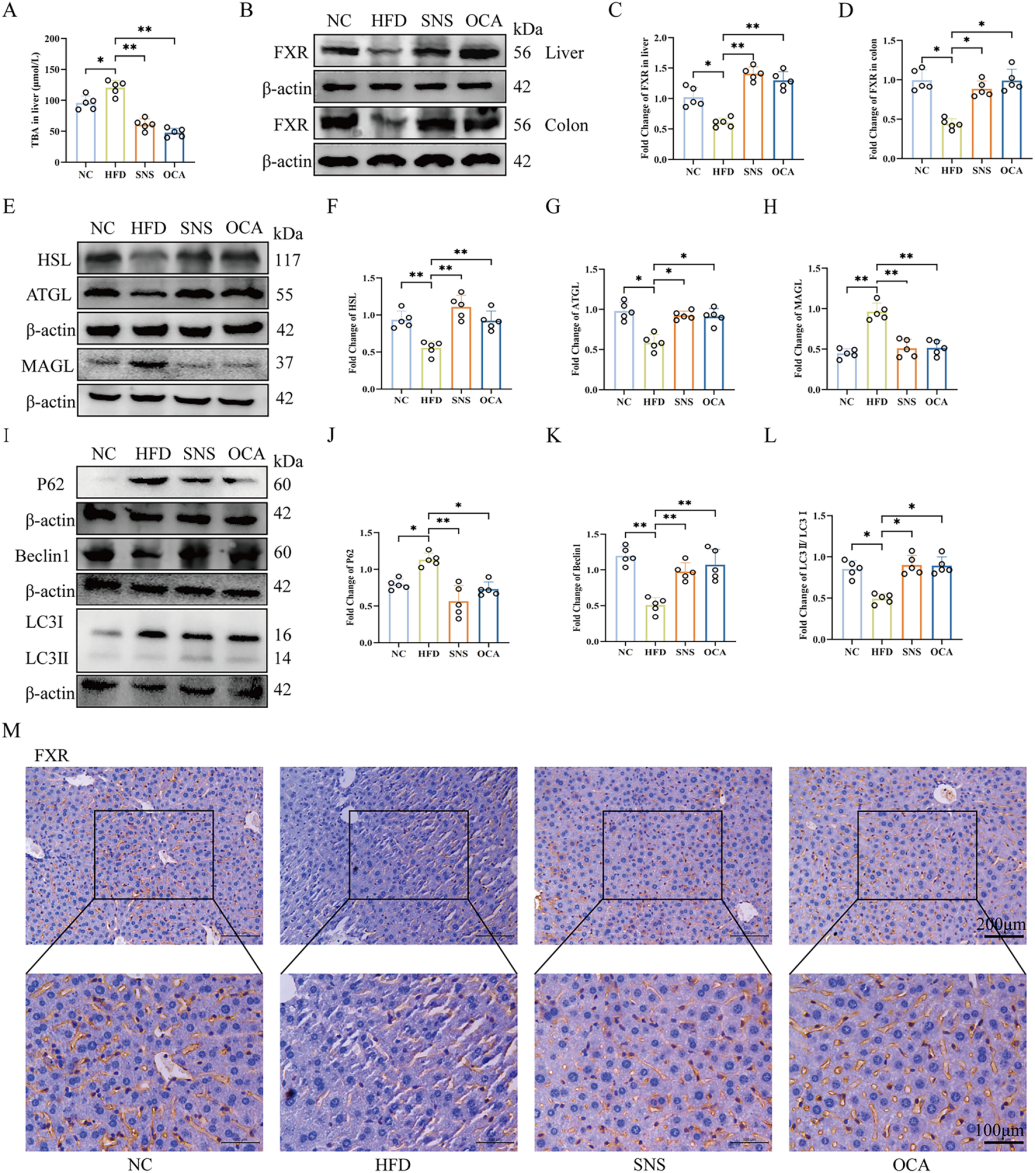
SNS activated FXR to regulate lipolysis- and lipophagy- related protein expression to improve MAFLD. **A** The level of TBA in the liver. **B**, **C**, **D** The expression levels of FXR protein in the liver and colon. **E**–**L** The expression level of proteins related to lipolysis and lipophagy in the liver. **M** IHC shows the expression level and localization of FXR in liver tissues. FXR is positively localized in hepatic stellate cells and hepatic sinusoids. Scale bar, 100 μm and 200 μm. Data are presented as mean ± SD (n = 5), *p < 0.05. **p < 0.01

To investigate the molecular mechanism by which SNS improves MAFLD, the TBA levels in the MAFLD mice were measured. HFD significantly elevated hepatic TBA levels, while SNS and OCA treatments reduced TBA levels (Fig. 4A). Since FXR is a bile acid receptor, hepatic FXR expression was also analyzed. HFD suppressed hepatic FXR expression, but SNS and OCA treatments activated its expression (Fig. 4B, D). FXR in the colon also plays a critical role in MAFLD. In our study, FXR expression in the colon of HFD group mice was reduced, whereas SNS and OCA treatments elevated colonic FXR expression (Fig. 4B–C). Meanwhile, IHC showed that SNS and OCA increased the positive localization of FXR in liver tissues, and the localization of FXR was mainly concentrated in hepatic cell nuclei and hepatic sinusoidal epithelial cells (Fig. 4M). These results suggested that SNS probably alleviated MAFLD by activating FXR in both the liver and colon.

FXR activation by OCA prompted further investigation of lipolysis- and lipophagy-related proteins in the liver. SNS and OCA treatments increased HSL and ATGL expression and decreased MAGL expression, promoting lipolysis and improving MAFLD (Fig. 4E–H). Regarding lipophagy, SNS and OCA treatments suppressed P62 expression and activated Beclin1. Meanwhile The ratio of LC3Ⅱ/LC3Ⅰ reflects the degree of lipophagy, and both SNS and OCA upregulated this ratio. (Fig. 4I–L). These findings indicated that SNS and OCA improved MAFLD by activating hepatic FXR, which subsequently regulated lipolysis- and lipophagy- related protein expression.

### SNS activated FXR to modulate LDs transport

**Fig. 5:**
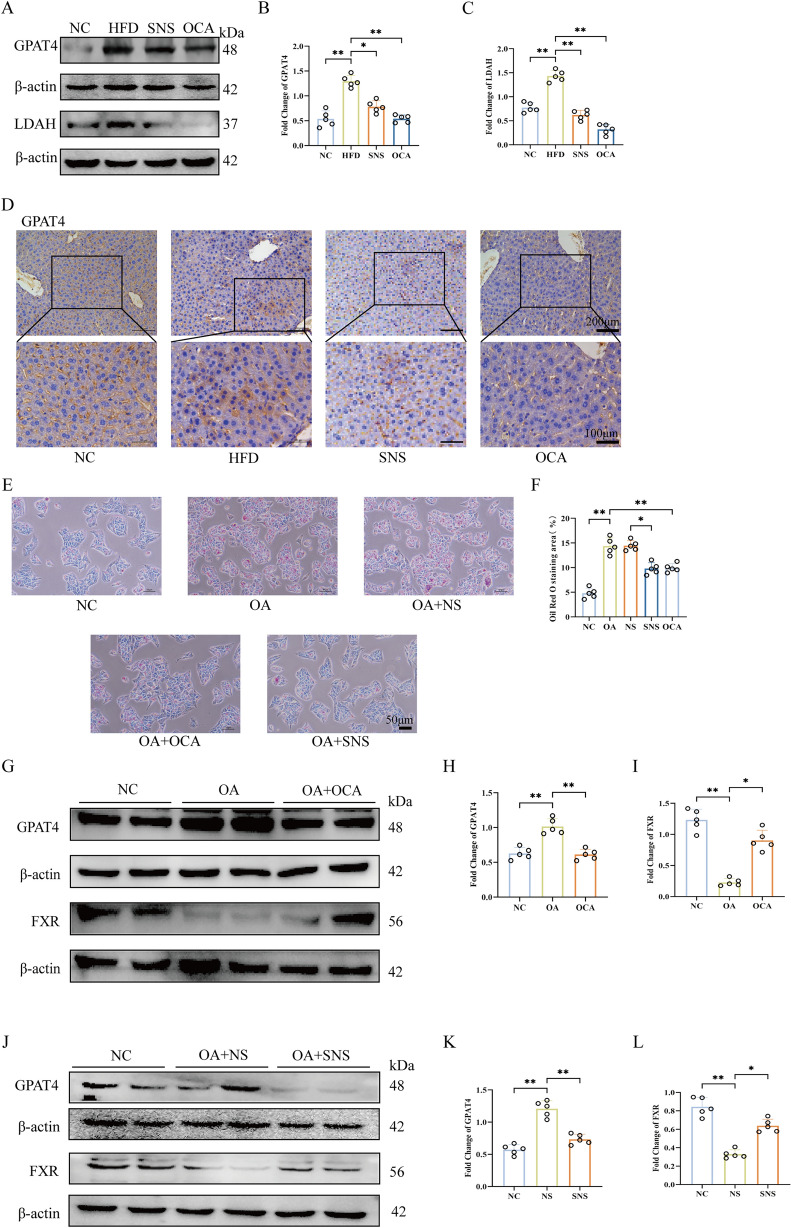
SNS activated FXR to modulate LDs transport. **A**–**C** Protein expression levels of GPAT4 and LDAH in liver tissues. **D** IHC staining demonstrates GPAT4 expression levels and localization in liver tissues. **E**, **F** ORO staining demonstrated successful model establishment, and SNS and OCA reduced LDs deposition in cells. **G**–**L** Western blot analysis was performed to confirm that SNS and OCA activated FXR and inhibited GPAT4 expression in vitro. Scale bars, 50 μm, 100 μm and 200 μm. Data are presented as mean ± SD (n = 5). *p < 0.05. **p < 0.01

Upon FXR activation, it may influence the targeting of proteins from the ER to LDs. Previous studies have identified multiple cargos involved in both early and late ER-to-LD targeting pathways. In this study, we focused on GPAT4, a late cargo responsible for ER-to-LD targeting [12]. HFD suppressed FXR expression while promoting GPAT4 expression. In contrast, SNS and OCA activated FXR and inhibited GPAT4 expressions (Fig. 5A–B). Meanwhile, the IHC showed that compared with the HFD group, SNS and OCA reduced the positive localization of GPAT4 in liver tissues, and the localization of GPAT4 was mainly concentrated in hepatic cytoplasm and hepatic sinusoidal epithelial cells (Fig. 5D). Although we did not systematically investigate the mechanism of ER-to-LD targeting early cargo, we preliminarily detected the protein level of LDAH in early cargo and found that both SNS and OCA also downregulated LDAH. This may suggest that the drugs also inhibit the early trafficking of LDs (Fig. 5A, C). This phenomenon was further validated in an MAFLD cell model. ORO staining showed that compared with NS serum, SNS serum significantly alleviated intracellular LDs accumulation, which was consistent with the results of OCA (Fig. 5E–F). Subsequently, the expression levels of FXR and GPAT4 in the cells were analyzed. Consistent with tissue results, SNS and OCA increased FXR expression and inhibited GPAT4 expression in cells (Fig. 5G–L). However, the precise regulatory mechanism linking FXR and GPAT4 requires further investigation.

### Knockdown of GPAT4 reduced LDs deposition, while knockdown of FXR increased GPAT4 expression

**Fig. 6:**
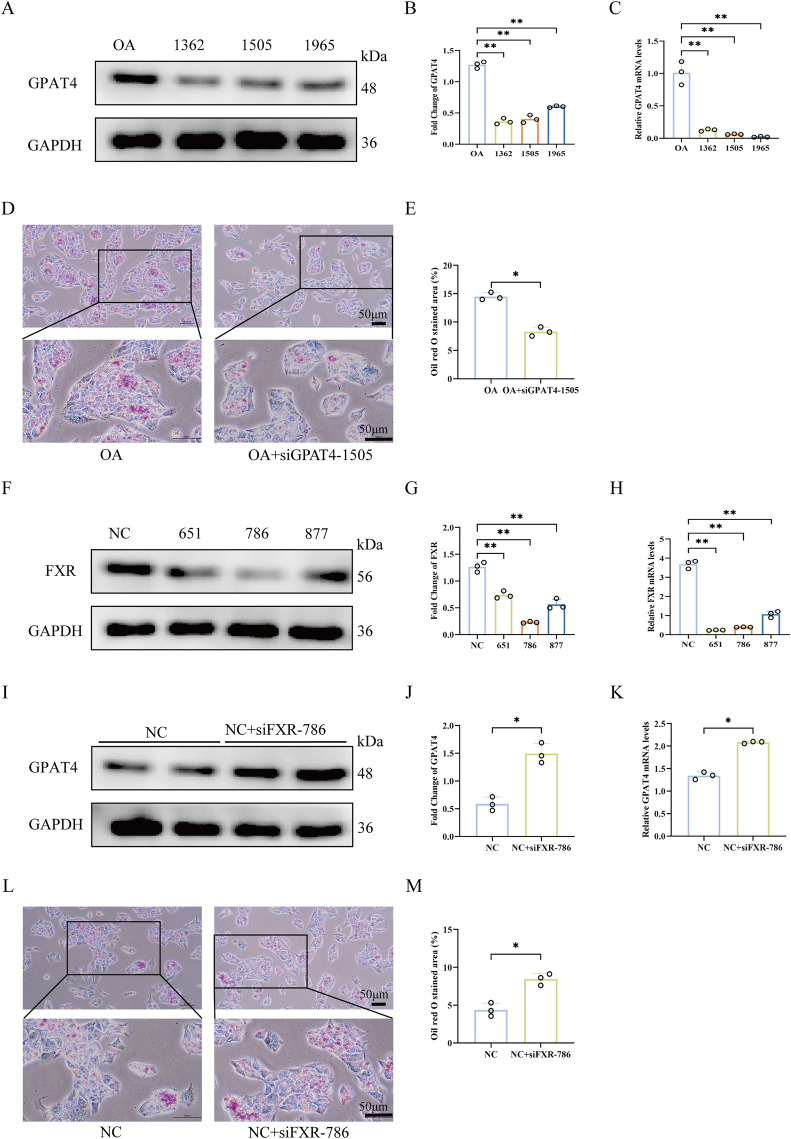
Knockdown of GPAT4 reduced LDs deposition, while knockdown of FXR increased GPAT4 expression. **A**, **B** The knockdown efficiency of GPAT4 was detected by Western blot assay. **C** The knockdown efficiency of GPAT4 was verified by PCR experiments, and 1505 was selected as the effective GPAT4 knockdown for subsequent experiments. **D**, **E** ORO staining was used to analyze the effect of GPAT4 knockdown on LDs deposition in cells. **F**, **G**, **H** Western blot and PCR experiments were used to screen and verify the knockdown efficiency of FXR, and 786 was selected for subsequent experiments. **I**, **J**, **K** After knocking down FXR, western blot and PCR experiments were performed to detect the expression level of GPAT4. **L**, **M** ORO staining was used to analyze the effect of FXR knockdown on LDs deposition in cells. Scale bar, 50 μm. Data are expressed as mean ± SD (n = 3). *p < 0.05. **p < 0.01

To investigate the role of FXR and GPAT4 in cellular LDs accumulation, siRNA targeting FXR (siRNA-FXR) was transfected into normal HepG2 cells, while siRNA-GPAT4 was transfected into oleic acid-induced HepG2 cells. First, the knockdown efficiency of GPAT4 was verified and 1505 was selected as the effective GPAT4 knockdown for subsequent experiments (Fig. 6A–C). ORO staining of cells showed that after effective knockdown of GPAT4 by transfection with 1505, the positive area of LDs deposition was reduced compared with the OA group (Fig. 6D–E). Meanwhile, the knockdown efficiency of FXR was verified and 786 was selected as the effective FXR knockdown for subsequent experiments (Fig. 6F–H). Subsequently, Western blot and PCR experiments were performed to detect the expression level of GPAT4 in cells after FXR knockdown. The results showed that the expression level of GPAT4 was upregulated after the FXR knockdown. Combined with the finding that GPAT4 expression was inhibited after FXR activation, it’s concluded that there is a close relationship between FXR and GPAT4, and transcriptional regulation may be involved (Fig. 6I–K). Then, ORO staining showed that FXR knockdown led to an increase in LDs deposition in cells. Considering that the inhibition of FXR may induce the development of MAFLD (Fig. 6L–M). These findings suggested a close relationship between FXR and GPAT4. As GPAT4 is involved in LDs transport, its inhibition may represent a novel therapeutic approach for improving MAFLD.

### Database predictions of FXR binding to GPAT4, validated by DL

**Fig. 7:**
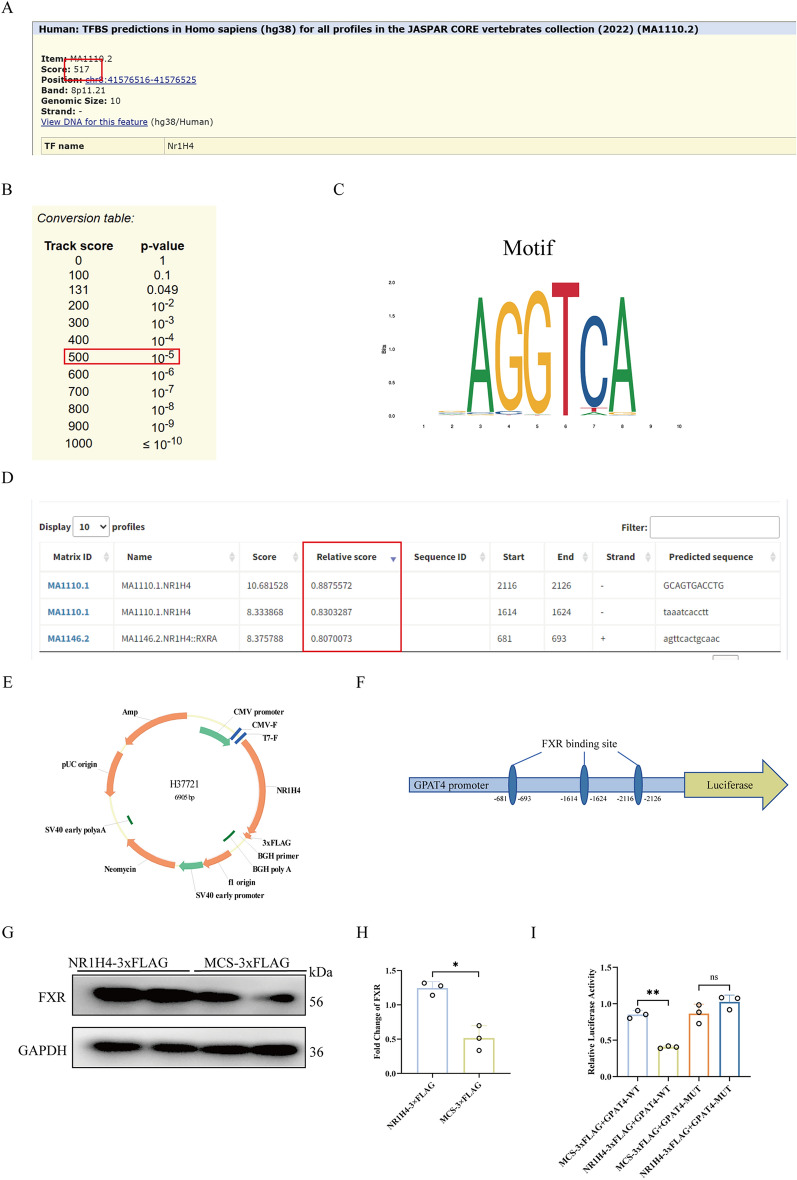
Database predictions of FXR binding to GPAT4, validated by DL. **A**, **B** The UCSC database was used to predict correlation scores and their corresponding p-values. **C** Predicted binding sequence of FXR. **D** The JASPAR database analyzed the binding correlation and potential binding sites between FXR and GPAT4. **E** Schematic diagram of constructing the plasmid for overexpressing FXR. **F** Schematic diagram of FXR binding sites in the three predicted GPAT4 promoter regions. **G**, **H** Western blot was used to detect the expression level of FXR in cells transfected with the plasmid overexpressing FXR. **I** DL was used to detect the binding of transcription factor FXR to the GPAT4 promoter region. Data are presented as mean ± SD (n = 3). *p < 0.05. **p < 0.01

To investigate the direct regulatory role of FXR on GPAT4, the GPAT4 promoter region sequence was retrieved from the NCBI database. The JASPAR database predicted a correlation and binding site between the GPAT4 promoter and the transcription factor FXR, the binding site located at positions 2116–2126,1614–1624, and 681–693 in the promoter region (Fig. 7D). Similarly, predictions using the UCSC database showed that FXR was the transcription factor most strongly correlated with the GPAT4 promoter, with a binding score of 419 and *p* ≤ 0.01 (Fig. 7A–B). The Schematic diagram of the sequence in the FXR binding element as shown in Fig. 7C. These results revealed there’s a potential binding interaction between FXR and the promoter region of GPAT4.

Then, the different plasmids were constructed and transfected into cells (Fig. 7E–F). Western blot analysis showed that FXR expression in cells transfected with the plasmid overexpression was higher than that in the empty vector group (Fig. 7G–H). Finally, a DL was performed to detect the luciferase activity values of the binding between the FXR overexpression plasmid/empty vector and the wild-type/mutant GPAT4 promoter regions, respectively. The results showed that in the wild-type group, the luciferase activity of FXR overexpression was lower than that of the empty vector group. However, after mutation of the GPAT4 promoter binding site, there was no significant difference in luciferase activity between the FXR overexpression and empty vector groups. These findings suggest that FXR binds to GPAT4 and inhibits its expression, while mutation of the binding site abolishes this interaction, leading to no significant difference in expression (Fig. 7I). This also indicated that SNS may ameliorate LDs deposition in MAFLD hepatocytes through the FXR-GPAT4 axis.

### The active components of SNS could bind to FXR and GPAT4

**Fig. 8:**
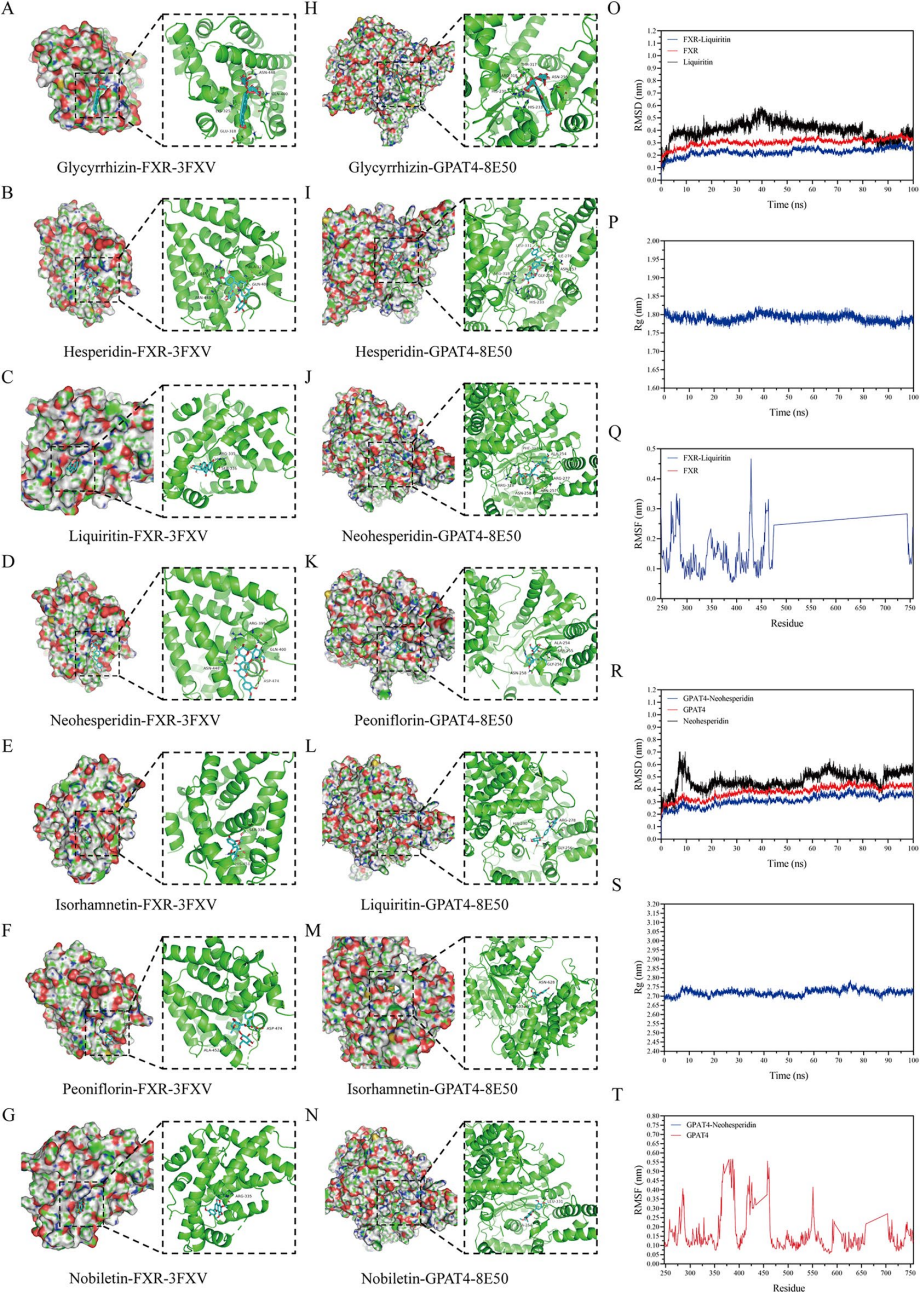
The active components of SNS could bind to FXR and GPAT4. **A**–**G** Visualization of the binding of Glycyrrhizin, Hesperidin, Liquiritin, Neohesperidin, Isorhamnetin, Peoniflorin and Nobiletinto to FXR. **H**–**N** Visualization of the binding of Glycyrrhizin, Hesperidin, Liquiritin, Neohesperidin, Isorhamnetin, Peoniflorin and Nobiletinto to GPAT4. **O**–**Q** The RMSD, Rg and RMSF values of the FXR-Liquiritin complex over time. **R**–**T** The RMSD, Rg and RMSF values of the GPAT4-Neohesperidin complex over time

To further investigate the binding of active components in SNS to FXR and GPAT4, these components were then subjected to molecular docking with FXR and GPAT4, respectively. The largest cavity identified on GPAT4 overlaps with the binding pocket of GPAT1, and three major cavities and multiple minor cavities on the GPAT1 structure (PDB ID: 8E50) were experimentally confirmed as intact binding sites. The binding energies of SNS active components with FXR and GPAT4 are presented in Table 4. First, the binding energies of all active components in SNS with FXR and GPAT4 were less than −7 kcal/mol, indicating a favorable binding affinity. Among them, glycyrrhizin showed the lowest binding energies with FXR (−14.7 kcal/mol) and GPAT4 (−13.1 kcal/mol), indicating the strongest binding affinity. Second, liquiritin showed a binding energy of −9.2 kcal/mol with FXR, while neohesperidin and esperidin displayed binding energies of −9.2 kcal/mol and −9.0 kcal/mol with GPAT4, respectively. These values, all less than −9 kcal/mol, suggest relatively stronger binding of liquiritin to FXR and of neohesperidin/hesperidin to GPAT4. Subsequently, molecular docking visualization was performed for each of these seven active components with FXR and GPAT4, respectively (Fig. 8A–N).

**Table 4:** Results of molecular docking

Key targets	PDB ID	Active ingredient	Binding energy (kcal/mol)
FXR	3FXV	Neohesperidin	−8.8
		Peoniflorin	−8.1
		Hesperidin	−8.9
		Glycyrrhizin	−14.7
		Liquiritin	−9.2
		Isorhamnetin	−8.2
		Nobiletin	−7.6
GPAT4	8E50	Neohesperidin	−9.2
		Peoniflorin	−8.9
		Hesperidin	−9.0
		Glycyrrhizin	−13.1
		Liquiritin	−8.6
		Isorhamnetin	−7.8
		Nobiletin	−7.4

Subsequently, molecular dynamics simulations were conducted to further observe the binding stability between small-molecule active components and target proteins. Among them, glycyrrhizin showed the highest binding affinity to FXR and GPAT4 (with binding energies of −14.7 kcal/mol and −13.1 kcal/mol). However, since the 3D structure of glycyrrhizin is unavailable, liquiritin (ranked second) was selected for molecular dynamics simulation of its binding to FXR, and neohesperidin (ranked second) was chosen for simulation of its binding to GPAT4. Additionally, both liquiritin-FXR and neohesperidin-GPAT4 bindings involve approximately 4–5 hydrogen bonds, indicating good stability. Both the FXR system and the FXR-liquiritin complex system reached equilibrium after 5 ns, with final fluctuations around 0.3 nm and 0.22 nm, respectively. The liquiritin small molecule reached equilibrium after 70 ns, with final fluctuations around 0.39 nm. Thus, the liquiritin small molecule exhibits high stability when binding to the target protein FXR. Both the GPAT4 system and the GPAT4-neohesperidin complex system reached equilibrium after 10 ns, with final fluctuations around 0.4 nm and 0.33 nm, respectively. The neohesperidin small molecule reached equilibrium after 10 ns, with final fluctuations around 0.55 nm. Therefore, the neohesperidin small molecule also shows high stability when binding to the target protein GPAT4 (Fig. 8O–R). The FXR-liquiritin complex showed relatively stable fluctuations during movement, suggesting no significant expansion or contraction of the small molecule-target protein complex. The GPAT4-Neohesperidin complex system exhibited slight fluctuations during movement, indicating conformational changes in the small molecule-target protein complex (Fig. 8P–S). The RMSF values of the FXR-liquiritin complex were relatively low (mostly below 0.3 nm), and those of the GPAT4-Neohesperidin complex were also relatively low (mostly below 0.4 nm), indicating low flexibility and high stability (Fig. 8Q–T). In summary, the complex systems exhibited stable binding with favorable hydrogen bond interactions. Therefore, the small molecules bind well to the target proteins.

### Verification of the binding between active components of SNS and FXR by SPR

**Fig. 9:**
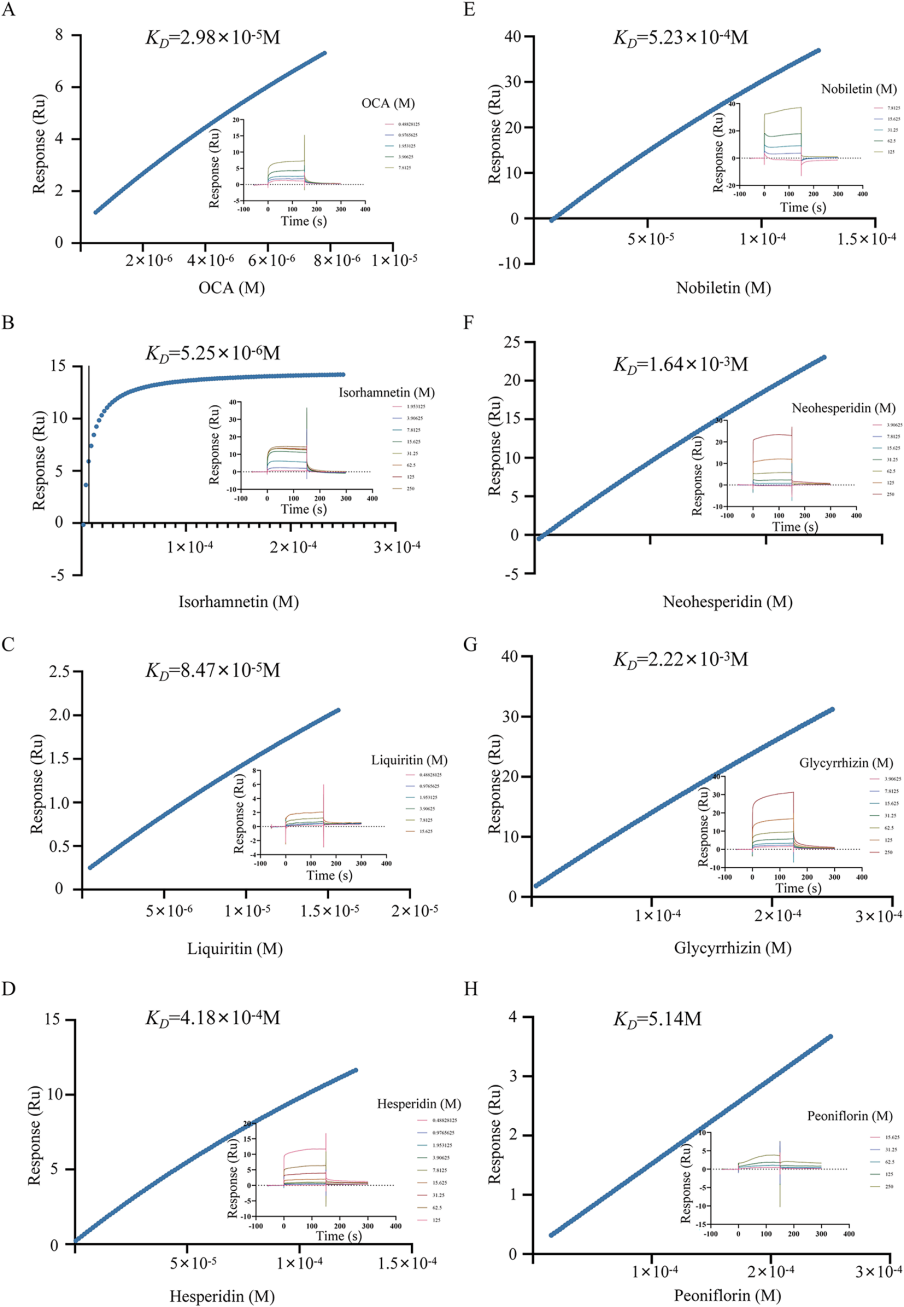
Verification of the binding between active components of SNS and FXR by SPR **A-H** SPR assays for the bindings of FXR to OCA, Isorhamnetin, Liquiritin, Hesperidin, Nobiletin, Neohesperidin, Glycyrrhizin and Peoniflorin. The insets depicted the surface plasmon resonance sensorgram of OCA, Isorhamnetin, Liquiritin, Hesperidin, Nobiletin, Neohesperidin, Glycyrrhizin and Peoniflorinat at various concentrations

Furthermore, SPR was used SPR to further substantiate the binding affinity between the compounds and the FXR, with the affinity reflected by the K_D_ value as shown in Table 5. The SPR analysis indicated that OCA displayed a favorable affinity for FXR, with an affinity constant of 2.98 × 10^−5^ M. This binding affinity was observed to be concentration-dependent (Fig. 9A). Among the other seven active ingredients, isorhamnetin exhibited a notable affinity for FXR (5.25 × 10^−6^ M), surpassing that of known agonists (OCA) and indicating its potential as a novel ligand for FXR activation (Fig. 9B). Liquiritin also demonstrated a favorable affinity for FXR (8.47 × 10^−5^ M), while molecular docking analysis suggested a high binding energy (Fig. 9C). Furthermore, hesperidin and nobiletin were found to have moderate affinity for FXR, with affinity constants of 4.18 × 10^−4^ M and 5.23 × 10^−4^ M, respectively (Fig. 9D–E). In contrast, neohesperidin and glycyrrhizin exhibited lower affinity for FXR, with affinity constants of 1.64 × 10^−3^ M and 2.22 × 10^−3^ M, respectively (Fig. 9F–G). Finally, Peoniflorin exhibits a very low affinity for FXR (5.14 M), suggesting that binding is highly unlikely (Fig. 9H). Therefore, isorhamnetin and liquiritin displayed favorable affinity for FXR, showing binding properties that are comparable to or surpass those of OCA. Both compounds exhibited concentration-dependent enhancements in binding affinity.

**Table 5:** The affinities of small molecules for FXR

No	Analytes	K_D_ (M)	Rmax (RU)	Chi^2^ (RU^2^)
1	OCA	2.98e-05	32.0	0.0123
2	Isorhamnetin	5.25e-06	20.3	1.18
3	Liquiritin	8.47e-05	12.1	0.00727
4	Hesperidin	4.18e-04	49.9	0.0381
5	Nobiletin	5.23e-04	210.1	2.13
6	Neohesperidin	1.64e-03	181.5	0.00762
7	Glycyrrhizin	2.22e-03	295.2	0.0685
8	Peoniflorin	5.14	73670.8	0.016

## Discussion

MAFLD is now one of the most common chronic liver diseases worldwide, with its incidence rising significantly due to links to obesity, diabetes, and metabolic syndrome—affecting about 25–30% of the general population and over 50% in high-risk groups. It could progress to severe liver conditions like fibrosis, cirrhosis, and hepatocellular carcinoma, and is linked to extrahepatic issues such as cardiovascular and metabolic disorders. Additional contributing factors to MAFLD include gut microbiota dysbiosis and genetic predisposition. Management primarily focuses on lifestyle changes—such as a low-sugar, low-fat, high-fiber diet and at least 150 min of weekly moderate aerobic exercise—along with pharmacological interventions like metformin for insulin resistance, vitamin E for antioxidant effects, statins for lipid regulation, and potential antifibrotic agents like pentoxifylline [25]. Meanwhile, Resmetirom (approved by the FDA for MASH treatment) and Semaglutide (approved for T2DM treatment) have also shown certain potential in improving hepatic lipid metabolism. Resmetirom, an FDA-approved oral THR-β agonist for MASH, and Semaglutide, approved for T2DM, have shown promise in ameliorating hepatic lipid metabolism. By boosting mitochondrial β-oxidation, Resmetirom reduces liver fat and fibrosis; notably, in the MAESTRO-NASH Phase III trial, it achieved 26–30% NASH resolution, 24–26% fibrosis improvement, and a 13–16% LDL-C decrease. The treatment was well-tolerated, with primarily mild diarrhea and nausea, and a serious adverse event rate comparable to placebo [26]. As a GLP-1 receptor agonist, Semaglutide promotes insulin secretion, inhibits glucagon, and delays gastric emptying, resulting in at least 10% weight loss and improved glycemia. It is also associated with an 18–20% reduction in major adverse cardiovascular events, as confirmed in trials such as SUSTAIN 6 and SELECT involving T2DM and high-risk obese individuals. The safety profile is characterized by common gastrointestinal reactions and infrequent serious adverse events [27]. Therefore, the management of MAFLD necessitates a comprehensive and individualized approach aimed at enhancing prognosis and reducing the risk of complications.

BAs regulate lipid metabolism in MAFLD via the FXR pathway, which suppresses hepatic lipogenesis genes to reduce triglyceride synthesis. They may interact with TGR5 to activate anti-inflammatory pathways and modulate cytokines, while FXR also inhibits hepatic stellate cell activation to mitigate fibrosis [28]. Additionally, BAs and gut microbiota interact bidirectionally—BAs alter microbial composition, and microbial metabolites modify BA metabolism—jointly influencing MAFLD pathogenesis and progression [29]. In our study, we examined the expression of FXR in both the liver and colon and observed that SNS influenced both. However, this study primarily focused on the hepatic FXR mechanism of action. The limited exploration of other mechanisms related to bile acid metabolism and MAFLD constitutes a limitation of this research.

FXR is a key target in MAFLD research, with its activation alleviating the disease through multiple mechanisms. By activating the FXR, OCA can significantly inhibit hepatic fibrosis and improve metabolic abnormalities in animal models and early-phase clinical trials. It is currently the only drug approved for bile duct liver diseases and granted breakthrough therapy designation in the field of MASH. Data from the FLINT and REGENERATE trials demonstrate its clear advantages in fibrosis improvement; however, its efficacy in achieving complete reversal of NASH remains limited by dosage and safety considerations [30]. It enhances bile acid transport genes like BSEP to promote hepatic bile acid excretion, reducing toxic accumulation and liver injury, while downregulating lipogenic enzymes (ACC, FAS) to lower triglyceride synthesis and hepatic fat [31, 32]. Following the conditional approval for the treatment of primary biliary cholangitis (PBC), the drug has been retracted from the European Union market by the European Medicines Agency (EMA). Additionally, Intercept Pharmaceuticals has voluntarily removed it from the United States market. Consequently, OCA is no longer accessible in these regions.

Additionally, FXR modulates inflammation by suppressing pro-inflammatory cytokines (TNF-α, IL-6) and inhibits hepatic stellate cell activation to exert anti-fibrotic effects, slowing liver fibrosis progression [33]. Despite these insights, the precise mechanisms by which hepatic FXR ameliorates MAFLD continue to be the subject of ongoing research.

Activating hepatic FXR effectively improves MAFLD, and this research will further explore its mechanisms. The TCM SNS shows promise in treating MAFLD by regulating lipid metabolism, improving insulin sensitivity, and reducing liver lipid deposition—its effects involve decreasing YAP1, activating AMPK to inhibit p300, reducing SREBP-1c stability, and suppressing Fasn to lower hepatocyte lipid accumulation [19, 34]. Additionally, SNS may enhance lipid metabolism via the AKT/AMPK/HSL axis and counteract stress-related MAFLD through the AMPK/SIRT1 pathway [35].

ATGL, HSL, and MAGL are key lipid-metabolizing enzymes—ATGL initiates triglyceride hydrolysis, HSL further degrades products, and MAGL acts on monoacylglycerols—collectively regulating lipid storage and mobilization in adipose tissue and liver [36]. Autophagy-related proteins P62 (selective degradation), Beclin1 (autophagy initiation), and LC3Ⅱ (autophagosome marker) are vital for cellular homeostasis and impact lipid metabolism and liver health [37]. FXR activation modulates the expression/activity of ATGL, HSL, and MAGL to affect lipid catabolism/synthesis, and influences autophagic flux by regulating P62-substrate interactions, Beclin1 activation, and LC3Ⅰ-to-LC3Ⅱ conversion [38]. FXR activation modulates the expression/activity of ATGL, HSL, and MAGL to affect lipid catabolism/synthesis, and influences autophagic flux by regulating P62-substrate interactions, Beclin1 activation, and LC3Ⅰ-to-LC3Ⅱ conversion.

GPAT4, a key triglyceride synthesis enzyme, contributes to MAFLD through abnormal overexpression that causes excessive hepatocellular triglyceride accumulation and disrupts LDs dynamics, while LDHA affects MAFLD progression by altering glycolysis, energy metabolism, and redox state to impact LDs transport and hepatic steatosis. Additionally, GPAT4 and LDHA act as late and early cargos for lipid transport from the endoplasmic reticulum to nascent LDs. In our research, it has been indicated that FXR can regulate GPAT4 and LDHA to improve MAFLD [12]. This study focused on GPAT4 based on these results. LDAH and GPAT4 are two proteins that target the ER to LD, influencing the transport of proteins from the ER to LDs, thereby reducing LDs synthesis. This research discussed the regulation of GPAT4 by FXR, yet the exploration of the mechanism regarding LDAH was not in depth.

Our research investigated SNS and FXR agonist OCA in improving MAFLD, finding they had similar effects: activating hepatic FXR altered expressions of lipolysis- and lipophagy-related proteins (HSL, ATGL, MAGL, p62, LC3Ⅱ, Beclin1), inhibited LDs transporters GPAT4 and LDHA, with FXR acting as an upstream transcriptional inhibitor of GPAT4 to reduce lipid accumulation. Additionally, SNS serum and OCA were effective in HepG2 cells, and inhibiting FXR in normal cells promoted lipid production, offering a potential clinical prevention idea for MAFLD.

This study explored SNS’s multi-component mechanism via HPLC and molecular docking, identifying seven active components (including neohesperidin, peoniflorin, and glycyrrhizin) with known roles in alleviating MAFLD-related processes like lipid accumulation and inflammation [39]. All components showed strong binding affinity to FXR and GPAT4 (binding energies < −7 kcal/mol), with Glycyrrhizin exhibiting the lowest, Liquiritin (to FXR) and Neohesperidin/Hesperidin (to GPAT4) showing high selectivity, supported by stable binding in molecular dynamics simulations. These findings indicate SNS acts through multi-component synergistic targeting of FXR and GPAT4, laying a foundation for its material basis research pending further in vitro and in vivo validation. SPR analysis demonstrated that active constituents in SNS, specifically isorhamnetin and liquiritin, exhibited a higher binding affinity for the FXR compared to OCA. These findings suggested that these compounds may serve as potential novel agonists for FXR, pending thorough evaluation of their pharmacological efficacy and toxicity profiles.

IHC of liver showed FXR and GPAT4 were prominently localized in hepatic sinusoids and vascular epithelial cells, with slight but insignificant co-localization in cells. This localization provides new insights into hepatic sinusoids’ role in LDs metabolism while highlighting the study’s limitations. Additionally, questions about how FXR localization affects LDs regulation, sinusoidal substance exchange’s relationship with lipid metabolism, and FXR’s role in these processes merit further exploration.

The DL studies gene expression regulation by fusing a gene’s promoter to a luciferase reporter and co-transfecting with a transcription factor vector; luciferase activity changes indicate the transcription factor’s binding ability and regulatory effect on the promoter. This study used DL to establish that FXR transcriptionally inhibits GPAT4, confirming their direct upstream–downstream regulatory relationship, though cell IF showed no significant co-localization specificity. With mutations at three predicted sites, FXR may act as GPAT4’s direct upstream regulator, but their specific binding sites require further exploration.

However, this study has some limitations. The dosage of TCM used in this study was calculated based on the conversion of body surface area between adults and mice, and since this study aimed to further explore the mechanism following the previous articles of our research group, no gradient of Chinese medicine dosage was designed. Meanwhile, this study screened the active components in the aqueous decoction of SNS; in subsequent studies, we will continue to detect the active components in SNS-containing serum and conduct in vivo and in vitro experimental studies on these active components.

## Conclusion

**Fig. 10:**
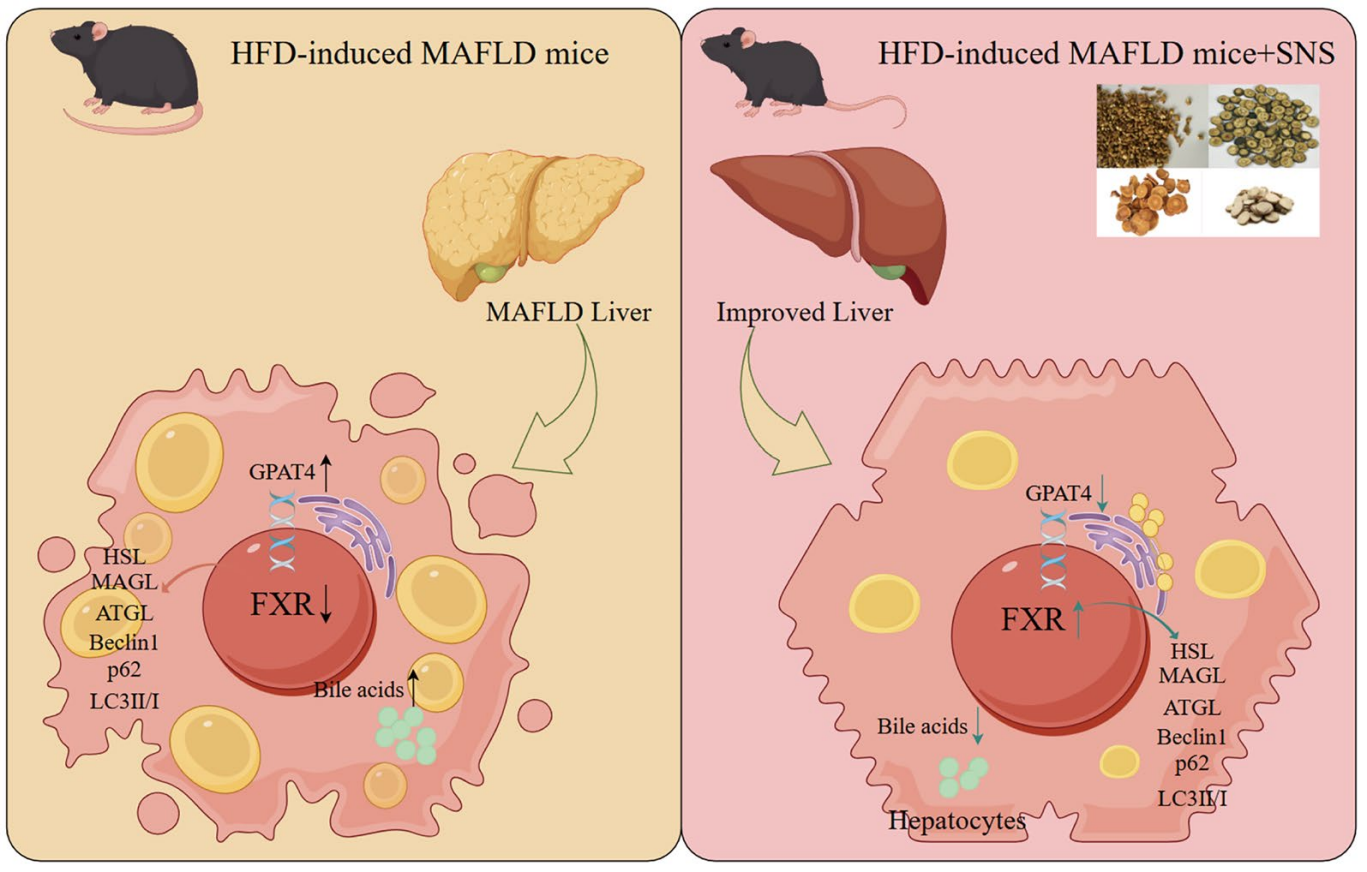
As illustrated in the figure, SNS activates hepatic FXR, inhibits the LDs transport protein GPAT4, and modulates the expression of proteins involved in lipolysis and lipophagy, including P62, Beclin1, LC3Ⅰ/Ⅱ, HSL, ATGL, and MAGL. Activation of hepatic FXR inhibits GPAT4 expression. Conversely, when FXR is inhibited, LDs deposition in liver cells worsens, leading to feedback upregulation of GPAT4 expression. The FXR-GPAT4 axis was validated using a DL

This study verified that SNS could effectively ameliorate LDs deposition in both the MAFLD mouse and cell models. It explored the mechanism by which SNS improved MAFLD through the FXR-GPAT4 axis and preliminarily investigated that the active components in SNS could bind to FXR and GPAT4, thus providing a mechanism innovation and experimental data support for the improvement of MAFLD by SNS (Fig. 10).

## Data Availability

The datasets supporting this study’s conclusions are accessible through the corresponding author upon a reasonable request.

## Abbreviations

MAFLD: Metabolic-associated fatty liver disease
SNS: Si-Ni-San
HFD: High-fat diet
FXR: Farnesoid X receptor
GPAT4: Glycerol 3-phosphate acyltransferase 4
HPLC: High-performance liquid chromatography
SPR: Surface plasmon resonance
TCM: Traditional Chinese Medicine
LDs: Lipid droplets
BAs: Bile acids
TG: Triglyceride
TC: Total cholesterol
ALT: Alanine aminotransferase
AST: Aspartate aminotransferase
HDL-C: High-density lipoprotein cholesterol
LDL-C: Low-density lipoprotein cholesterol
OCA: Obeticholic acid
ATGL: Adipose triglyceride lipase
MAGL: Monoacylglycerol lipase
HSL: Hormone-sensitive lipase
P62: Sequestosome 1
LC3: Microtubule-associated protein 1 light chain 3
SPF: Specific pathogen-free
ORO: Oil red O
H&E: Hematoxylin and eosin staining
IHC: Immunohistochemistry assay
DL: Dual-luciferase reporter assays
RMSD: Root mean square deviation
RMSF: Root mean square fluctuation
Rg: Radius of gyration
MD simulation: Molecular dynamics simulation
DMSO: Dimethyl sulfoxide
